# Fatal drowning in older adults with alcohol, opioids, cannabinoids, and other drug exposures

**DOI:** 10.1093/geroni/igaf122.812

**Published:** 2025-12-31

**Authors:** Armiel Suriaga, Jessica Mangila, Hien Do, Lindsey Gensoli, Grace Abrams, Karina Del Carpio, Alondra Gittleson, Gianna Nemia

**Affiliations:** Florida Atlantic University, Boca Raton, Florida, United States; Boston University, Boston, Massachusetts, United States; Florida Atlantic University, Boca Raton, Florida, United States; Florida Atlantic University, Boca Raton, Florida, United States; Florida Atlantic University, Boca Raton, Florida, United States; Florida Atlantic University, Boca Raton, Florida, United States; Florida Atlantic University, Boca Raton, Florida, United States; Florida Atlantic University, Boca Raton, Florida, United States

## Abstract

More than 4,500 people die annually from drowning in the U.S., with a 19% increase among 65+ in 2022 compared to 2019. However, research on drowning-related mortality (DRM) among older adults exposed to alcohol, opioids, cannabis, and other substances is scarce. We aimed to report the characteristics of older adults who drowned with substances, using de-identified data from the Florida Department of Law Enforcement (2020-2022). We used descriptive statistics and regression modeling in Stata 17. The medical examiners ascertained the outcome (drowning) through autopsy and toxicology results. The analysis included 46,437 decedents with substances, 1,162 (2.5%) decedents with DRM, and 265 (4.64%) were older adults. 187 (70.57%) of the 265 were males, white= 228 (86.04%), Black=28 (10.57%), and 252 (95.1%) occurred in urban counties. 177 (66.79%) used a single drug and 88 (33.21%) with polydrug exposures. 70 (26.42%) had cardiac-related conditions, 16 (6.04%) with respiratory, and 15 (5.66%) had neurocognitive-related conditions. Older adults have lower odds of drowning than the reference group (0-17), OR=.68, 95% CI, .48-.95, p = 0.03. Those with alcohol had 3.26 higher odds of drowning compared to those without alcohol, OR = 3.26, 95% CI, 2.88-3.70, p < 0.001; opioids, OR=.19, 95% CI, .16-.22, p < 0.001; cannabinoids, OR=.85, 95% CI, .73-.99, p = 0.04; benzodiazepine, OR=.70, 95% CI, .59-.81, p < 0.001, and stimulant, OR=.73, 95% CI, .61-.87, p < 0.001. Polydrug exposures were significantly associated, OR=.42, 95% CI, .38-48, p < 0.001. Our results suggest that using multiple substances such as alcohol, opioids, and cannabis increases the odds of DRM, underscoring the need for tailored interventions to safeguard older adults.

